# Effectiveness of Insulin Analogs Compared with Human Insulins in Pregnant Women with Diabetes Mellitus: Systematic Review and Meta-analysis

**DOI:** 10.1055/s-0038-1676510

**Published:** 2019-02

**Authors:** Leyna Leite Santos, Jamilly Leite Santos, Luciano Timbó Barbosa, Ivan do Nascimento da Silva, Célio Fernando de Sousa-Rodrigues, Fabiano Timbó Barbosa

**Affiliations:** 1 Universidade Federal de Alagoas, Maceió, AL, Brazil; 2 Universidade Federal de Pernambuco, Recife, PE, Brazil; 3 Hospital Geral do Estado Professor Osvaldo Brandão Vilela, Maceió, AL, Brazil; 4 Centro Universitário CESMAC, Maceió, AL, Brazil

**Keywords:** diabetes mellitus, gestational diabetes, human insulin, insulin analogs, pregnancy, diabetes mellitus, diabetes gestacional, insulina humana, análogos da insulina, gestação

## Abstract

Diabetes during pregnancy has been linked to unfavorable maternal-fetal outcomes. Human insulins are the first drug of choice because of the proven safety in their use. However, there are still questions about the use of insulin analogs during pregnancy. The objective of the present study was to determine the effectiveness of insulin analogs compared with human insulin in the treatment of pregnant women with diabetes through a systematic review with meta-analysis. The search comprised the period since the inception of each database until July 2017, and the following databases were used: MEDLINE, CINAHL, EMBASE, ISI Web of Science, LILACS, Scopus, SIGLE and Google Scholar. We have selected 29 original articles: 11 were randomized clinical trials and 18 were observational studies. We have explored data from 6,382 participants. All of the articles were classified as having an intermediate to high risk of bias. The variable that showed favorable results for the use of insulin analogs was gestational age, with a mean difference of - 0.26 (95 % confidence interval [CI]: 0.03–0.49; *p* = 0.02), but with significant heterogeneity (Higgins test [I^2^] = 38%; chi-squared test [χ^2^] = 16.24; degree of freedom [DF] = 10; *p* = 0.09). This result, in the clinical practice, does not compromise the fetal well-being, since all babies were born at term. There was publication bias in the gestational age and neonatal weight variables. To date, the evidence analyzed has a moderate-to-high risk of bias and does not allow the conclusion that insulin analogs are more effective when compared with human insulin to treat diabetic pregnant women.

## Resumo

Diabetes durante a gestação tem sido relacionado a desfechos materno-fetais desfavoráveis. As insulinas humanas são a primeira escolha medicamentosa, devido à comprovada segurança no seu uso. Entretanto, ainda há questionamentos sobre o uso dos análogos da insulina na gestação. O objetivo do presente estudo foi determinar a efetividade dos análogos da insulina comparados às insulinas humanas no tratamento de gestantes com diabetes por meio de uma revisão sistemática com metanálise. A busca compreendeu desde o início de cada base de dados até julho de 2017, e foi realizada nos seguintes bancos de dados: MEDLINE, CINAHL, EMBASE, ISI Web of Science, LILACS, Scopus, SIGLE e Google Scholar. Selecionamos 29 artigos originais, sendo 11 ensaios clínicos randomizados e 18 estudos observacionais. Exploramos dados de 6.382 participantes. Todos os artigos foram classificados como sendo de intermediário a alto risco de viés. A variável que demonstrou resultado favorável ao uso dos análogos da insulina foi idade gestacional, com uma diferença média de - 0.26 (95% índice de confiança [IC]: 0.03–0.49; *p* = 0.02), porém com heterogeneidade significativa (teste de Higgins [I^2^] = 38%; teste do qui quadrado [χ^2^] =16.24; graus de liberdade [GL] =10; *p* = 0.09). Esse resultado, na prática clínica, não compromete o bem-estar fetal, uma vez que todos os bebês nasceram a termo. Houve viés de publicação nas variáveis idade gestacional e peso neonatal. Até o momento, as evidências analisadas possuem um risco de viés moderado a elevado e não permitem concluir que os análogos da insulina sejam mais efetivos em comparação às insulinas humanas para tratar gestantes diabéticas.

## Introduction

Diabetes mellitus (DM) is currently a serious public health issue. It may be estimated that there are 425 million adults with DM worldwide, with a projection of 629 million in 2045.^1^ Diabetes mellitus stands as the main metabolic complication of pregnancy, and may occur in two different clinical contexts: the woman has a previous diagnosis of diabetes (previous DM) or develops it during the pregnancy (gestational DM [GDM]).^2^

Persistent hyperglycemia is a harmful factor for all pregnancies. Still, patients with previous DM are in a more serious situation, since hyperglycemia may influence negatively, on pregnancy, since the period of fertilization and implantation.^3^ ^4^ Possible complications for the child are: congenital malformations, macrosomia, spontaneous abortion, perinatal asphyxia, traumas during childbirth, hypoglycemia, and respiratory distress syndrome, among others. The pregnant women may suffer from polyhydramnios, premature rupture of the amniotic membranes, premature birth, toxemia of pregnancy, higher occurrence rates of caesarean sections and mortality, and, in addition, the worsening of chronic complications of the already existing diabetes, such as retinopathy and nephropathy. Negative consequences may also occur in the long-term, both for the mother and the child, such as increased risk of obesity, glucose intolerance, and type 2 DM for the child. The mother, on the other hand, is more susceptible to a gestational DM relapse, dyslipidemia, type 2 DM, and systemic arterial hypertension.^3^ ^4^ ^5^ ^6^

It is of utmost importance to maintain the glycemic control during pregnancy, because this improves mother and fetal outcomes. Insulin therapy is considered the gold standard treatment, and the use of human insulins during this period is already well established. However, divergences still exist regarding the use of insulin analogs in this clinical situation due to conflicting results found on previous studies.^7^ ^8^

Thus, the focused question was: what is the effectiveness of insulin analogs compared with human insulins for the treatment of pregnant women with diabetes?

The aim of the present systematic review was to determine the effectiveness of insulin analogs compared with human insulins for the treatment of pregnant women with diabetes.

## Methods

A systematic review with meta-analysis of original articles was performed to assess the use of insulin analogs for the treatment of diabetic pregnant women. The method of the present research followed the recommendations to perform systematic reviews proposed by the Cochrane Collaboration.^9^ The ROBIS tool was published in 2016 and serves to assess the risk of bias in systematic reviews. A recommendation, which is part of this tool, is to make the protocol available to the public or to disclose it after its registration in the database.^10^ In this way, a protocol was developed a priori and is available by contacting the authors, if the public shows interest. The present study was conducted according to the recommendations of the Preferred Reporting Items for Systematic Review and Meta-Analyses (PRISMA).^11^

### Eligibility Criteria

Original articles of randomized clinical trials (RCTs) and observational studies (cohort and case-control) that used human insulins and insulin analogs for the treatment of pregnant women with diabetes (GDM and previous DM) were included in the present study. The following were excluded: duplicated papers; articles with incomplete description of data regarding research development; studies with inadequately described interventions; and studies with a population that carried any type of diabetes other than type 1, type 2, or GDM.

### Outcomes

The primary outcomes of the present systematic review were: maternal glycemic control (fasting blood glucose and glycated hemoglobin), congenital malformation, fetal death, and maternal death. The secondary outcomes were: macrosomia, gestational age, abortion, neonatal weight, neonatal hypoglycemia, and maternal hypoglycemic episodes.

### Search Strategy and Study Selection

The following online databases were used without restrictions of date, language, or any other: MEDLINE, CINAHL, EMBASE, ISI Web of Science, LILACS, Scopus, SIGLE, and Google Scholar. The search was performed since the inception of each database until July 2017. References of included papers were also screened. The search strategies were adapted according to the rules of each database and are available on Table 1.

**Table 1:** Search strategy

Databases	Search Strategy
PUBMED	*diabetes, gestational* [MeSH Terms] OR (*diabetes* [All Fields] AND *gestational* [All Fields]) OR *gestational diabetes* [All Fields] OR (*gestational* [All Fields] AND *diabetes* [All Fields]) AND *insulin, isophane* [MeSH Terms] OR (*insulin* [All Fields] AND *isophane* [All Fields]) OR *isophane insulin* [All Fields] OR (*nph* [All Fields] AND *insulin* [All Fields]) OR *nph* *insulin* [All Fields]
EMBASE	*NPH insulin* OR *glargine* OR *lispro* OR *aspart* AND *gestational* *diabetes*
LILACS	(*gestational diabetes*)
CINAHL	*NPH insulin* OR *glargine* OR *lispro* OR *aspart* AND *gestational diabetes*
SCOPUS	(*diabetes*) AND (*gestation*) AND (*insulin*) OR (*nph*) OR (*glargine*) OR (*lispro*) OR (*aspart*) OR (*regular* AND *insulin*)
ISI Web of Science	*NPH insulin* OR *Glargine* OR *Lispro* OR *Aspart* AND *Gestational Diabetes*
GOOGLE SCHOLAR	*pregnancy,,* *insulin*
SIGLE	(*gestational diabetes*)

### Statistical Analysis

Qualitative data of the included studies were reviewed, and, when possible, quantitatively combined using the Review Manager (RevMan), Version 5 software (Cochrane Collaboration, Copenhagen, Denmark). A random effects model was used. Dichotomous data were calculated by means of relative risk (RR) with a 95% confidence interval (CI). Relative risk differences (RRD) with a 95% CI were used if an event on the outcome did not occur. Continuous outcomes were analyzed through standardized mean difference with a 95% CI. The significance level was set at 5%. An inverted funnel plot was used to detect publication bias.

### Sensitivity Analysis

The sensitivity analysis was performed considering the risk of bias. The Newcastle Ottawa Quality Assessment Scale (NOS) was used as a quality assessment tool for observational studies. Results from observational studies with a score of eight or nine stars on NOS were assessed separately from the other observational studies. Regarding RCTs, the risk of bias table (RBT) of the Cochrane Collaboration was used. The RCTs classified as having a low risk of bias would be compared with studies that received the lower classification of the risk of bias in at least one of the criteria of the risks of biases.

### Homogeneity Analysis

We have used the following tests that are available for meta-analyses graphs in the RevMan version 5 software to assess statistical heterogeneity: 1) Chi-squared test (χ^2^); 2) *p*-value; 3) Degree of freedom (DF); and 4) Higgins test (I^2^). An I^2^ ≥ 50% was adopted for the present systematic review as a significant value and as a representative of the heterogeneity among studies. This method was indicated as the main factor for the assessment of statistical heterogeneity among studies. Heterogeneities, when identified, were analyzed considering the individual characteristics of each paper. A meta-regression analysis would be performed only if there were 10 or more studies and when I^2^ was > 50%.

## Results

### Original Articles Identified

**Fig. 1:**
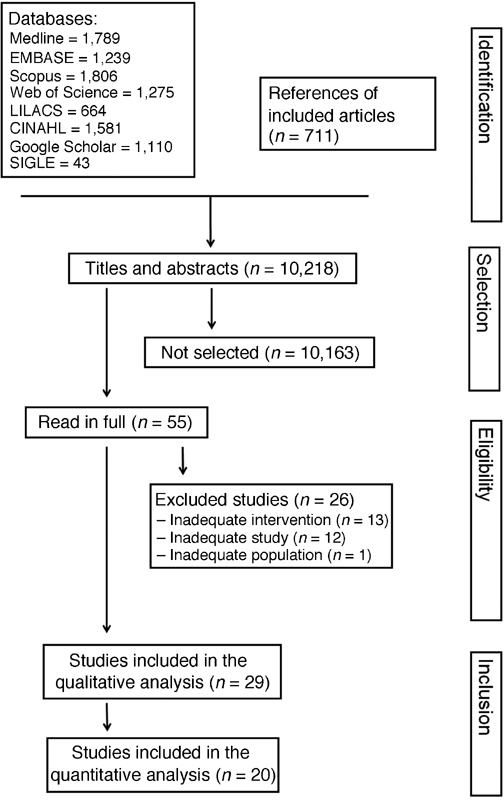
Flowchart showing the search results of the data sources, of the selection, and of the inclusion of original articles in the systematic review.

The number of original articles identified on each database, as well as the number of included and excluded original papers on the present systematic review, are presented on Fig. 1.^12^ ^13^ ^14^ ^15^ ^16^ ^17^ ^18^ ^19^ ^20^ ^21^ ^22^ ^23^ ^24^ ^25^ ^26^ ^27^ ^28^ ^29^ ^30^ ^31^ ^32^ ^33^ ^34^ ^35^ ^36^ ^37^ ^38^ ^39^ ^40^ Twenty-nine original articles were selected through search strategies, of which 11 were randomized clinical trials and 18 were observational studies. Data from 6,382 participants were analyzed. The characteristics of the included studies are shown on Table 2.

**Table 2:** Characteristics of the included studies

Author, year of publication	Type of study	Pregnant women treated with insulin	Human insulin	Insulin analog	Type of Diabetes	Conclusions
Aydin, 2008^12^	Observational	86	Regular	Lispro	Previous DM + GDM	Congenital anomalies were more frequent with lispro and similar with regular insulin, but HbA1c was lower. Other outcomes were similar.
Balaji, 2012^13^	RCT	320	Premixed human 30 (BIH 30)	Premixed aspart 30 (BIAsp 30)	GDM	BIAsp 30 was not inferior than BIH 30, with comparable fetal results. Based on final dosages, BIAsp 30 may offer a better potential of treatment.
Banerjee, 2009^14^	Observational	153	Regular	Lispro	Previous DM	Lispro provides a better glycemic control and does not adversely affect maternal and fetal results.
Bhattacharyya, 2001^15^	Observational	220	Regular	Lispro	Previous DM + GDM	No increase in the adverse results was found with Lispro insulin. The satisfaction of the patients favored Lispro.
Chico, 2010^16^	Observational	315	Regular	Lispro	Previous DM	Lispro was independently associated with less hypoglycemic comas. Its impact on the fetus was favorable or unfavorable, depending on the specific result.
Chico, 2016^17^	Observational	1,210	NPH	Glargina	Previous DM	The type of base insulin was independently associated with metabolic outcomes and fetal endpoints.
Colatrella, 2013^18^	Observational	89	NPH	Insulin lispro protamine	Previous DM + GDM	The result with Lispro Protamine insulin was similar to NPH, except for a smaller need for insulin.
Cypryk, 2004^19^	Observational	71	Regular	Lispro	Previous DM	The course of pregnancy and perinatal results were comparable. Humalog seems to be a safe alternative to human insulin.
Dalfra, 2015^20^	Observational	933	NPH	Insulin lispro protamine	Previous DM + GDM	The association of ILPS with fast action analogs during pregnancy is safe regarding maternal and fetal results.
Durnwald, 2008^21^	Observational	107	Regular	Lispro	Previous DM	Lispro showed a better glycemic control and a smaller need for total insulin during pregnancy. Perinatal results were similar.
Egerman, 2009^22^	Observational	114	NPH	Glargine	Previous DM + GDM	Neonatal or maternal adverse effects were not observed with the use of Glargine insulin.
Fang, 2009^23^	Observational	112	NPH	Glargine	Previous DM + GDM	Glargine is not associated with the increase of maternal and neonatal morbidity. On previous DM, Glargine was associated with less macrosomia, hypoglycemia, and neonatal hyperbilirubinemia.
García-Dominguez, 2011^24^	Observational	351	Regular	Lispro and Aspart	Previous DM	Analogs are safe on previous DM. Glycemic control and maternal and fetal results were similar. Analogs significantly reduced severe hypoglycemia on the mother.
Heller, 2010^25^	RCT	223	Regular	Aspart	Previous DM	The beginning of treatment with insulin analogs at preconception, instead of at the beginning of pregnancy, may result in a smaller risk of severe hypoglycemia in women with DM1.
Herrera, 2015^26^	RCT	87	NPH	Detemir	Previous DM + GDM	Detemir is not inferior to NPH for the treatment of GDM and of DM2 during pregnancy.
Hod, 2008^27^	RCT	268	Regular	Aspart	Previous DM	Fetal outcome using Aspart was comparable to human insulin with a trend to less fetal losses and premature births.
Hod, 2014^28^	RCT	310	NPH	Detemir	Previous DM	Detemir is as well tolerated as NPH regarding perinatal results in pregnant women with DM1, with no safety issues.
Imbergamo, 2008^29^	Observational	30	NPH	Glargine	Previous DM	There was no significant difference in the glycemic control between glargine and NPH insuline. Use of glargine was associated with a significantly higher frequency of femoral length < 50th centile.
Jovanovic, 1999^30^	RCT	42	Regular	Lispro	GDM	Lispro insulin may be considered as a treatment option for women with GDM.
Lapolla, 2008^31^	Observational	370	Regular	Lispro	Previous DM	There was a trend for less episodes of hypoglycemia in the Lispro group and also a significant reduction on HbA1c during the first trimester. The congenital malformation rates were similar.
Loukovaara, 2003^32^	Observational	69	Regular	Lispro	Previous DM	Lispro insulin improves the glycemic control during pregnancy with no adverse impact on the progression of diabetic retinopathy.
Mathiesen, 2007^33^	RCT	322	Regular	Aspart	Previous DM	Aspart is as safe and effective as human insulin when in base therapy with NPH insulin and may offer some benefits for postprandial glycemic control and for the prevention of severe hypoglycemia.
Mathiesen, 2012^34^	RCT	310	NPH	Detemir	Previous DM	Treatment with detemir resulted in lower FPG and noninferior A1C in late pregnancy compared with NPH insulin. Rates of hypoglycemia were comparable.
Negrato, 2010^35^	Observational	138	NPH	Glargine	Previous DM + GDM	The use of glargine since preconception until birth showed safety because it is associated with a reduction in adverse maternal and neonatal outcomes when compared with NPH.
Persson, 2002^36^	RCT	33	Regular	Lispro	Previous DM	It is possible to obtain a proper glycemic control with Lispro, as well as with regular insulin, in pregnant women with DM1.
Pettit, 2007^37^	RCT	27	Regular	Aspart	GDM	Aspart was more effective than regular insulin in the reduction of postprandial glycemia. General safety and effectiveness were similar.
Price, 2007^38^	Observational	64	NPH	Glargine	Previous DM + GDM	The use of Glargine during pregnancy may not be associated with an increase of macrosomia and of neonatal morbidity.
Pöyhönen-Alho, 2007^39^	Observational	91	NPH	Glargine	Previous DM	Glargine is comparable to NPH on DM1. No adverse effect was associated with Glargine on the moment of conception and during pregnancy.
Vellanki, 2016^40^	RCT	87	NPH	Detemir	Previous DM + GDM	Detemir is effective and did not increase the risk of fetal or maternal adverse outcomes on GDM and previous DM.

Abbreviations: DM, Diabetes Mellitus; GDM, Gestational Diabetes Mellitus; HbA1c, hemoglobin A1c; NPH, Neutral protamine Hagedorn; RCT, randomized clinical trial.

Source: Santos, 2018.

Regarding the insulin analog used, 7 studies used glargine, 4 studies used detemir, 2 used lispro protamine, 10 used lispro, 4 used aspart, 1 used premixed aspart 30, and 1 assessed the use of lispro or aspart. Of the populations studied, 16 studies included only pregnant women with previously diagnosed DM, 3 articles analyzed only women with GDM, and 10 articles studied pregnant women with GDM and previous DM.

**Fig. 2:**
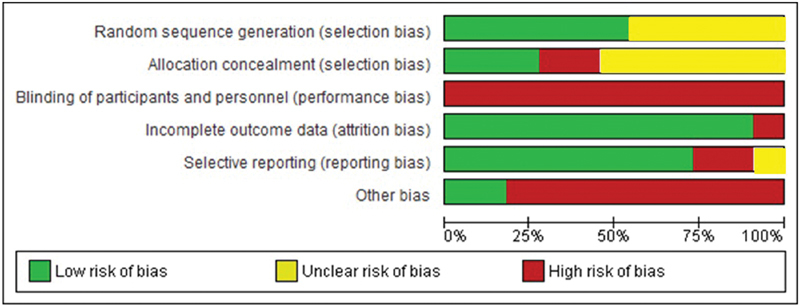
Methodological quality assessment for all RCTs according to the criteria of the risk of bias table of the Cochrane Collaboration.

The methodological quality assessment showed that RCTs as well as observational studies were classified as having a moderate or high risk of bias. No study was classified as having a low risk of bias (Fig. 2).

### Outcomes Assessed

**Fig. 3:**
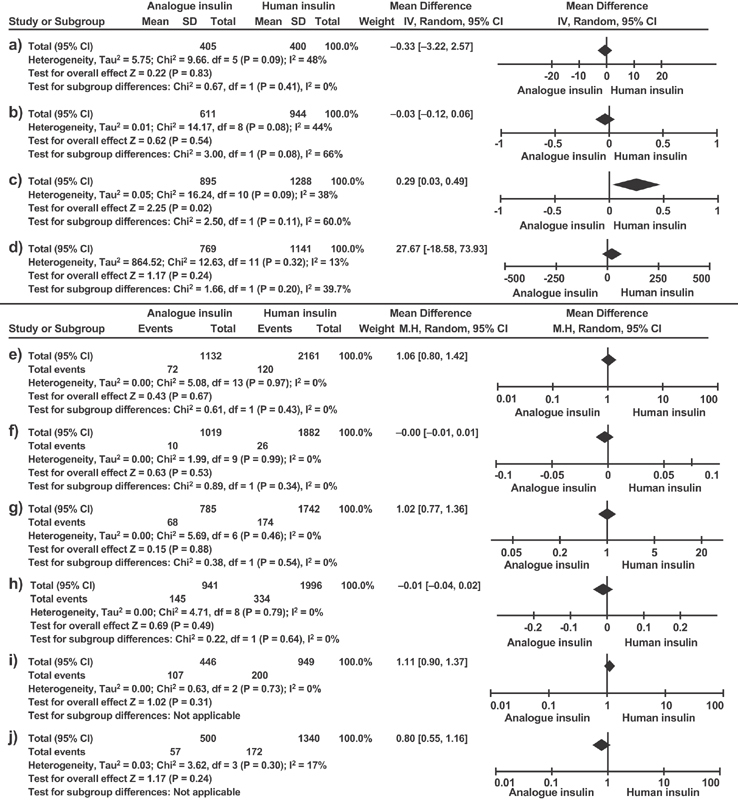
Meta-analyses of all the outcomes studied. Meta-analyzes a) fasting glycemia; b) glycated hemoglobin; c) gestational age; d) neonatal weight; e) congenital malformation; f) perinatal mortality; g) abortion; h) macrosomia; i) neonatal hypoglycemia; j) maternal hypoglycemia.

**Maternal glycemic control – blood glucose:** This variable was reported on 14 original papers. However, only 6 studies were included on this meta-analysis.^13^ ^18^ ^25^ ^26^ ^29^ ^40^ The mean difference of blood glucose was of - 0.33 (95% CI: -3.22–2.57; *p* = 0.83; 1,619 participants). There was statistical heterogeneity on this comparison (I^2^ = 48%; χ^2^ = 9.66; DF = 5; *p* = 0.009) (Fig. 3A). Publication bias was not identified.

**Maternal glycemic control – glycated hemoglobin:** This variable was analyzed on 26 original papers; 9 of them contributed to this meta-analysis.^13^ ^18^ ^19^ ^22^ ^24^ ^25^ ^29^ ^31^ ^39^ The mean difference of hemoglobin A1c (HbA1c) was of -0.03 (95% CI: - 0.12–0.06; *p* = 0.54; 1,555 participants). Statistical heterogeneity was present on this comparison (I^2^ = 44%; χ^2^ = 14.17; DF = 8; *p* = 0.08) (Fig. 3B). Publication bias was not detected on this analysis.

**Congenital malformation:** This variable was described on 19 original articles. However, 14 participated on this meta-analysis.^12^ ^16^ ^17^ ^18^ ^19^ ^21^ ^22^ ^24^ ^27^ ^28^ ^29^ ^31^ ^36^ ^39^ The mean difference was of 1.06 (95% CI: 0.80–1.42; *p* = 0.67; 3,293 participants). Statistical heterogeneity was not present on this comparison (I^2^ = 0%; χ^2^ = 5.08; DF 13; *p* = 0.97) (Fig. 3E). Publication bias did not exist for this outcome.

**Perinatal mortality:** This variable was analyzed on 11 original papers. Only one paper was not included on this meta-analysis.^35^ The mean difference of this outcome was of 0.00 (95% CI: - 0.01–0.01; *p* = 0.53; 2,901 participants). Statistical heterogeneity was not present on this comparison (I^2^ = 0%; χ^2^ = 1.99; DF = 9; *p* = 0.53) (Fig. 3F). It can be observed that publication bias did not occur for this outcome.

**Maternal death:** this outcome was not reported on the original articles. Therefore, the meta-analysis could not be performed.

**Macrosomia:** This variable was described on 12 original articles, 9 of them had adequate data for this meta-analysis.^12^ ^13^ ^16^ ^17^ ^21^ ^24^ ^28^ ^31^ ^37^ The mean difference of this outcome was of - 0.01 (95% CI: - 0.04–0.02; *p* = 0.49; 2,937 participants). Statistical heterogeneity did not exist for this comparison (I^2^ = 0%; χ^2^ = 4.71; DF = 8; *p* = 0.79) (Fig. 3H). Publication bias was not detected.

**Gestational age:** This variable was reported on 22 included original articles, of which only 12 contributed to this meta-analysis. The mean difference of this outcome was of 0.26 (95% CI: 0.03–0.49; *p* = 0.02; 2,183 participants). There was statistical heterogeneity on this comparison (I^2^ = 38%; χ^2^ = 16.24; DF = 10; *p* = 0.09) (Fig. 3C). There was publication bias for this outcome. When the studies with suspected bias were withdrawn, the analysis was performed with the results from Aydin et al (2008)^12^ and of Hod et al (2008),^27^ and the statistical result changed, showing no difference between insulins.

**Abortion:** was analyzed on seven original articles, of which only one reported results inadequately. The mean difference of this outcome was of 1.02 (95% CI: 0.77–0.36; *p* = 0.88; 2,527 participants). Statistical heterogeneity did not exist for this comparison (I^2^ = 0%; χ^2^ = 5.69; DF = 6; *p* = 0.46) (Fig. 3G). There was no occurrence of publication bias.

**Neonatal weight:** This variable was described on 23 original papers. However, 12 of them were used for this meta-analysis.^12^ ^13^ ^18^ ^19^ ^21^ ^22^ ^24^ ^28^ ^29^ ^31^ ^37^ ^39^ The mean difference of this outcome was of 27.67 (95% CI: - 18.58–73.93; *p* = 0.24; 1,910 participants). Heterogeneity was present, but it was not statistically significant on this comparison (I^2^ = 13%; χ^2^ = 12.63; DF = 11; *p* = 0.32) (Fig. 3D). Publication bias existed on this analysis due to the inclusion of the results of Durnwald et al (2008).^21^ The statistical result did not change when this study was withdrawn.

**Neonatal hypoglycemia:** was reported on 20 original papers, but only 3 observational studies were used,^17^ ^19^ ^22^ because the concepts and descriptions of this outcome varied among authors. The mean difference of this outcome was of 1.11 (95% CI: 0.90–1.37; *p* = 0.31; 307 participants). Statistical heterogeneity did not occur for this comparison (I^2^ = 0%; χ^2^ = 0.63; DF = 2; *p* = 0.73). (Fig. 3I). There was no occurrence of publication bias.

**Maternal hypoglycemia:** This variable was reported on 20 original articles, but we have used data from only 4 observational studies for this meta-analysis,^16^ ^17^ ^24^ ^29^ due to differences in the concept and in the description of this outcome among authors. The mean difference of this outcome was of 0.80 (95% CI: 0.55–1.16; *p* = 0.24; 1,840 participants). Heterogeneity existed, but it was not statistically significant (I^2^ = 17%; χ^2^ = 3.62; DF = 3; *p* = 0.30) (Fig. 3). There was no occurrence of publication bias.

### Sensitivity and Homogeneity Analysis of Included Studies

The sensitivity analysis was planned due to the risk of bias in the articles included in the present study. However, this analysis was not performed, since all of the eligible original articles were classified as having a moderate or high risk of bias. Regarding the cohort studies, the NOS score ranged from five to six stars, and regarding the case-control studies, the score ranged from three to six stars. None of them were classified as having a low risk of bias. Likewise, no RCT was classified as having a low risk of bias (Fig. 2). The sensitivity analysis was also not performed because it did not fit in a previously established criteria. The main flaw of the eligible studies was related to blinding, since all of them were open trials. Statistical bias existed mainly due to the absence of sample size calculation, in most cases.

## Discussion

Insulin is the drug of choice for the treatment of hyperglycemia during pregnancy, since it does not cross the placental barrier in significant amounts.^41^ The use of insulin analogs for the treatment of diabetic pregnant women is still being discussed, and it is not completely disseminated in the clinical practice, despite the evidence of its safety profile. Thus, there is still a preference for the use of human insulins on the gestational period.^42^ ^43^ ^44^

The need for the type of insulin to be used will vary progressively throughout the gestation, due to an increase in insulin resistance, which may begin with a dose of 0.5 U/kg.^44^ Generally, it is recommended the use of a smaller proportion of the total daily dose as base insulin (< 50%) and a higher proportion (> 50%) as prandial insulin. The scheme may be of multiple daily doses or of continuous infusion. Adjustments must be performed according to the self-monitoring of capillary blood glucose.^41^

The results analyzed on the present systematic review come from studies classified as having a moderate or high risk of bias. Therefore, the evidence generated on the present review can be classified as low. Consequently, this was the main limitation of the review process. Studies with a high risk of bias tend to present positive results that may lead reviewers to inadequate conclusions.^9^ A sensitivity analysis was planned, but it was not performed because the eligible articles were considered as having a moderate or high risk of bias.

Some reasons prevented the inclusion of all of the studies on the present meta-analysis, such as: the separation of participants in two different groups for analysis (previous DM and GDM); the different concepts reported for neonatal hypoglycemia and maternal hypoglycemia; and, in addition, the different ways of presenting results or the absence of gross results reported in the text. We have tried, unsuccessfully, to contact the authors to fill this blank.

Most of the eligible papers were observational studies (18 articles). These papers were different among themselves, which limits the use of their results in the clinical practice. To minimize this limitation and to explore potential sources of heterogeneity, we planned to perform a meta-regression. However, it was not possible to perform it because it did not fit in the criteria for this analysis.

Heterogeneity occurred on the analysis of the following outcomes: fetal weight, maternal hypoglycemia, glycemic control, and gestational age, with statistical significance on the last two. It was only possible to identify the studies responsible for heterogeneity through the method of successive withdrawals and inclusions of the outcome gestational age, which were the studies of Balaji et al (2012)^13^ and of Colatrella et al (2013).^18^

It is known that a high level of blood glucose is responsible for several complications that may affect both the mother and the fetus.^45^ ^46^ Therefore, is of utmost importance to reach blood glucose levels within the previously established marks in the literature. (HbA1c: 6–6.5%; fasting blood glucose: 95 mg/dL; postprandial blood glucose 1 hour: 140 mg/dL, and 2 hours: 120 mg/dL). The assessment of the fasting blood glucose and glycated hemoglobin levels is optional for this control.^40^ Blood glucose marks become increasingly stricter during pregnancy. Women identified with poor glycemic control should have a special attention from the health team to avoid maternal and fetal complications.

Regarding blood glucose level and glycated hemoglobin, the assessment of the final result of the present meta-analysis showed that the use of insulin analogs did not provide a better glycemic control compared with the use of human insulins. Analyzing the results from RCTs of the outcome glycated hemoglobin, the result favored the use of insulin analogs. Nevertheless, the RCTs were classified as having a moderate or high risk of bias. Still, there was no difference between the use of insulin analogs and the use of human insulins on maternal glycemic control.

Hyperglycemia during pregnancy may interfere on fetal organogenesis, leading to congenital abnormalities.^47^ ^48^ It remains unclear if the use of insulin analogs during pregnancy might increase the risk of congenital malformations. A population retrospective cohort study did not show the increase of congenital abnormalities in women with previous diabetes that were exposed to insulin analogs on the first trimester of pregnancy, with a significant reduction of congenital cardiac defects.^49^ The analysis of this outcome on the present systematic review showed that there is no higher damage on fetus formation when using insulin analogs. This can probably be explained by the fact that there is an insignificant transplacental passage of insulin, which does not result in direct damage to the fetus.^41^

Mortality after the 22nd week of gestational age or of the newborn’s is one of the possible complications of the occurrence of diabetes during pregnancy, which may affect between 2.8 and 6.2% of the newborns. Data from previous studies showed that the perinatal mortality and stillbirth rates were higher in pregnant women with DM1 than in the general population.^47^ ^50^ ^51^ Persistent fetal hyperinsulinemia results in higher oxygen uptake and metabolic rates, which consequently increase the risk of fetal hypoxemia and mortality.^52^ The analysis of this outcome showed that there was no difference between the use of insulin analogs and the use of human insulins on perinatal death. Since the passage of insulins through the placenta is not significant, the treatment of pregnant women with insulin analogs was not responsible for an increase in perinatal mortality rates.

Maternal death is one of the possible fatal complications of poorly controlled diabetes during pregnancy, which presented high occurrence rates in the past. The introduction of insulin therapy for the treatment of gestational hyperglycemia changed the history of pregnant women with diabetes. The eligible studies did not analyze this outcome. Hence, the statistical analysis wasn’t performed as planned. Therefore, it is not possible to know if the event did not happen in the studied groups or if it was not an outcome of interest in these researches. A possible explanation can also be a reduction in the occurrence of this event due to a better metabolic control in recent years, due to a more rigorous prenatal follow-up. Nevertheless, the answer to the focused question cannot be confirmed by the analysis of this outcome.

Persistent hyperglycemia may lead to excessive fetal growth, exceeding 4 kg. Thus, macrosomia is a frequent complication in pregnant women with diabetes, which is potentially harmful for both the mother and the fetus, with risk of traumas in childbirth, fetal hypoxia (and even neonatal death), perineal laceration, uterine atony, and severe hemorrhagy.^53^ Hyperglycemia is considered a changeable risk factor for macrosomia.^54^ On the present systematic review, there was no difference between the use of insulin analogs and the use of human insulins regarding macrosomia, reaffirming the importance of avoiding hyperglycemia during pregnancy.

In two important population studies, newborns from diabetic mothers were born at a significantly smaller gestational age when compared with control newborns. It was partially justified for policy of early induction of labor and maybe for poorly controlled diabetes.^47^ ^50^ The result of this outcome is statistically significant, showing that the gestational age at the time of childbirth is smaller with human insulin. However, in the clinical practice, this difference of days does not compromise the well-being of the fetus, since childbirth at > 37 weeks is considered as being at term.

Pregnant women with diabetes are more prone to fetal death before the 22^nd^ week of gestational age,^6^ ^55^ and pregnant women with a previous diagnosis of diabetes are even more prone to this complication.^3^ ^4^ The results of the present meta-analysis for this outcome did not show a difference regarding the therapy used. Therefore, the use of insulin analogs did not increase the risk of miscarriage. This fatal endpoint is probably related to uncontrolled maternal blood glucose, and not to the type of insulin used.

In diabetic gestations, a proper glycemic control allows a more satisfactory fetal growth. It was already shown that an excessive glycemic control was related to the increase in the incidence of smaller newborns for the gestational age, while the acceptance of more flexible levels of blood glucose resulted in larger newborns for the gestational age. Both situations are related to fetal complications.^56^

In a prospective study, the mean weight at birth was similar between newborns of diabetic mothers and of controls.^50^ Still, in a recent Swedish study performed between 1991 and 2003, the birth weight was higher in infants of diabetic mothers.^47^ Babies with higher birth weight have a higher risk of death.^57^ The analysis of this outcome did not show a difference in fetal weight between the use of insulin analogs and the use of human insulins. This shows that, regardless of the type of insulin used, the important thing is to reach the recommended glycemic marks, in order to avoid blood glucose extremes that may interfere in fetal growth.

The excess of glucose crosses the placental barrier and results in a hyperglycemic uterine environment. Consequently, the fetal pancreatic cells become hyperplasic, which leads to an increase in the secretion of insulin. In the afterbirth, fetal hyperinsulinism persists regardless of the end of maternal glucose. Hence, the risk of neonatal hypoglycemia increases and may cause severe neurological damage and even death.^58^ This is one of the most common complications in children of diabetic mothers, and may occur in between 10 and 50% of these gestations. A study that assessed the use of insulin analogs during pregnancy showed a high incidence of hypoglycemic babies at birth.^59^

Data from the studies included in the present meta-analysis revealed that there was no difference regarding the use of insulin analogs or of human insulins on the occurrence of neonatal hypoglycemia. However, the small amount of analyzed studies may have influenced these results.

Only a few observational studies participated in the present meta-analysis of the outcomes neonatal hypoglycemia^17^ ^19^ ^22^ and maternal hypoglycemia.^16^ ^17^ ^24^ ^39^ The exclusion of the other articles from the respective meta-analyses was mainly because a divergence existed regarding the definition of the outcomes among the studies. This conceptual difference made impossible to compare the studies.

Insulin analogs are considered as effective as human insulins on the treatment of diabetes; however, with a smaller risk of causing hypoglycemia. This could be explained by the absence (or reduction) of peak action of these analogs due to their stabler profile and smaller glycemic variability.^45^ ^60^ Nevertheless, the present systematic review revealed that insulin analogs did not seem to cause less maternal hypoglycemia compared with human insulins, which is in line with the findings of Siebenhofer et al (2006).^61^

The present systematic review presented publication bias in the outcomes gestational age and neonatal weight, and the suspected studies were then identified. After the withdrawal of the studies responsible for the publication bias on the outcome neonatal weight,^21^ the statistical result did not change. Regarding the outcome gestational age, the analysis with all articles favored human insulin. When the studies suspected of causing bias were withdrawn,^13^ ^18^ ^19^ ^21^ ^22^ ^24^ ^28^ ^29^ ^31^ ^39^ the statistical result changed and did not favor any type of insulin. Still, this does not interfere in the clinical practice because all babies were born at term.

The evidence from the present systematic review is insufficient to recommend the use of insulin analogs for all pregnant women with diabetes, due to the fact that the analyzed studies were classified as having a moderate or high risk of bias. The controversy persists, and there is not enough evidence to justify the universalization of insulin analogs as the standard therapy for all pregnant women with diabetes. The choice of the type of insulin rests with the specialist physician, who will take into consideration the social and clinical profile of the patient, the physiological effects of the chosen intervention associated to its benefits and possible risks, and the preferences and personal experiences of the patients.

The implication of the findings of the present systematic review for the clinical practice is the possibility of safely choosing the drug for the treatment of pregnant women with diabetes, in which case it is possible to use both human insulin and insulin analogs. Both choices are able to allow the achievement of a satisfactory glycemic control, without disagreement regarding the occurrence of hypoglycemia in the mother or in the baby, of fetal loss, of congenital malformations, or of inadequate neonatal weight. The use of human insulin results in childbirth at a smaller gestational age. However, these births occur at term, which does not compromise fetal vitality and, therefore, should not be taken into account in the choice of the medication. The difference in the type of insulin used will be basically related to the cost (lower with the human insulins) and to the posologic convenience (more comfortable with the insulin analogs).

Thus, to compose the drug therapy, neutral protamine Hagedorn (NPH), detemir, or glargine may be used as basal insulin. For the control of postprandial blood glucose, regular insulin, as well as lispro and aspart analogs are available. The adjustment of basal insulin will depend on fasting and preprandial glycemia. Since postprandial glycemia is elevated, fast-acting or ultra-rapid-acting drugs may be adjusted before the main meals from home monitoring of capillary glycemia.^62^ Maintaining metabolic control is the key to a favorable maternal-fetal outcome.

A systematic review with meta-analysis presents a more robust recommendation power when it explores the results from RCTs with low risk of bias. Thus, one may suggest for future researches that the RCTs be better designed, with reports of the sample size calculation, and with a proper randomization and blinding with allocation concealment. In this context, it is crucial to set uniform concepts for the outcomes of interest and to use the same therapeutic scheme of insulin therapy to reach a proper glycemic control.

## Conclusion

The present systematic review with meta-analysis showed that, to this point, the available evidences have a moderate or high risk of bias and cannot support the conclusion that insulin analogs are more effective when compared with human insulins for the treatment of diabetic pregnant women.
